# Accuracy of Anthropometric Equations to Estimate DXA-Derived Skeletal Muscle Mass in Professional Male Soccer Players

**DOI:** 10.1155/2019/4387636

**Published:** 2019-01-01

**Authors:** Roberto Gabriel González-Mendoza, Alejandro Gaytán-González, Juan Antonio Jiménez-Alvarado, Marisol Villegas-Balcázar, Edtna E. Jáuregui-Ulloa, Francisco Torres-Naranjo, Juan R. López-Taylor

**Affiliations:** ^1^Institute of Applied Sciences for Physical Activity and Sport, Department of Human Movement Sciences, Education, Sport, Recreation and Dance, University Health Sciences Center, University of Guadalajara, Guadalajara, JAL, Mexico; ^2^Department of Human Reproduction, Infantile Growth and Development, University Health Sciences Center, University of Guadalajara, Guadalajara, JAL, Mexico; ^3^Center of Body Composition and Bone Research, Guadalajara, JAL, Mexico

## Abstract

**Background:**

Several anthropometric equations that estimate skeletal muscle mass (SMM) have been published, but their applicability and accuracy among athletes are still uncertain.

**Purpose:**

To assess the accuracy of different anthropometric equations that estimate SMM in professional male soccer players, as compared to dual-energy X-ray absorptiometry (DXA) as the reference method.

**Methods:**

In this cross-sectional study, we evaluated 179 professional male soccer players aged between 18 and 37 years. Anthropometric measurements (height, body weight, skinfold thicknesses, and girths) and a DXA whole body scan were performed the same day for each participant, and SMM was estimated with nine anthropometric equations (Heymsfield, Martin, Doupe, Kerr, Drinkwater, Lee, De Rose, and two equations published by Kuriyan). To determine differences between SMM estimated with anthropometric equations and SMM evaluated with DXA, a Kruskal-Wallis test was performed using Dunn's test as post hoc. The significance level was set at* p* < 0.05. We calculated the mean difference and 95% limits of agreement for the analyzed equations (Equation – DXA).

**Results:**

Only Heymsfield's and Lee's equations showed no significant differences with DXA. Heymsfield's equation had the smallest mean difference (-0.17 kg), but wider limits of agreement with DXA (-6.61 to 6.94 kg). Lee's equation had a small mean difference (1.10 kg) but narrower limits of agreement with DXA (-1.83 to 4.03 kg).

**Conclusions:**

In this study, the prediction equation published by Lee et al. showed the best agreement with DXA and is able to estimate SMM accurately in professional male soccer players.

## 1. Introduction

Soccer is a demanding high intensity, intermittent sport [1, 2]. Players need to train to adapt to this type of stimuli with specific sport exercise programs. The desired adaptive-responses include increasing aerobic capacity, muscle strength, power, endurance, and increased skeletal muscle mass (i.e., skeletal muscle hypertrophy) (SMM) [1, 3]. SMM is associated with exercise performance and injury risk reduction [4, 5]. Therefore, the accurate assessment of SMM is important in the evaluation of soccer players [4, 6].

There are several methods to assess SMM; one of them is with dual-energy x-ray absorptiometry (DXA) [7]. Although DXA does not assess SMM directly, some studies have reported the potential to calculate the SMM from appendicular lean soft tissue (ALST) using DXA. Moreover, there are studies that have validated the estimation of SMM through DXA-derived ALST in adults [8] and athletes [9].

However, many sport-performance trainings facilities do not have access to DXA equipment; therefore other simpler methods, such as anthropometry, are used to evaluate SMM [10]. It is easier to conduct anthropometric measurements in the field, a feasible alternative compared to expensive imaging methods [10]. The anthropometric measurements are usually employed to estimate SMM through several equations [11–18], but most of them were developed in nonathletic populations. Some studies have analyzed the accuracy of these equations in college students [19] and in older adults [20]. To our knowledge, there are few studies regarding the accuracy of anthropometric equations in athletic populations, and among those that have been conducted, the results have been inconclusive [21–23]. Therefore, the purpose of this study was to compare anthropometric equations that estimate SMM with that derived from DXA in order to find which ones are more accurate in professional male soccer players.

## 2. Methods

### 2.1. Participants

We obtained data in a cross-sectional way from 179 professional male soccer players, aged 18 to 37 years. Subjects were evaluated from 2009 to 2018 as part of their annual medical assessment which took place in our laboratory at 9:00 a.m. after a 2-hour fast. They were instructed to avoid any exercise prior to their evaluations. These consisted of anthropometric measurements and a DXA whole-body scan performed on the same day. Only nonrepeated data were analyzed, and if multiple evaluations were registered, we kept the most recent. All subjects read and signed a written statement of consent prior to the evaluations.

### 2.2. Anthropometry

Measurements consisted of the assessment of body mass to the nearest 0.1 kg (Tanita TBF-410, Tanita Corporation of America Inc., Illinois, USA); height to the nearest 0.1 cm (SECA 214, SECA, Hamburg, Germany); 8 measures of skinfolds thickness (triceps, subscapular, biceps, iliac crest, supraspinal, abdominal, front thigh, and medial calf) to the nearest 0.2 mm (Harpenden, Baty International, West Sussex, United Kingdom); 11 girths (arm relaxed, arm flexed and tensed, forearm, wrist, chest, waist, gluteal [hip], thigh, mid-thigh, calf, ankle) to the nearest 0.1 cm (Lufkin W606PM, Lufkin, North Carolina, USA); and 3 bone breadths (biepicondylar humerus, bi-stylon, biepicondilar femur) to the nearest 1 mm (Campbell 10, Rosscraft, Canada). Trained personnel assessed all measurements following standardized protocols [24, 25]. The intraobserver technical error of measurement (TEM) in our laboratory is ≤5% for skinfolds and ≤1% for all other measurements, and the interobserver TEM is ≤7.5% for skinfolds and ≤1.5% for all other measurements.

### 2.3. DXA Scanning

A whole-body scan was performed for each subject with DXA fan beam equipment (Hologic QDR Explorer, Massachusetts, USA). The machine was calibrated daily according to the manufacturer's manual. To calculate bone mineral content and the fat and lean body mass, the scan was analyzed with a Hologic QDR v 12.1 software for Windows® (1986–2002©, Hologic Inc.), and the appendicular lean soft tissue (ALST) was obtained as described by Kim et al [8]. A certified DXA technician executed the whole procedure. The difference between DXA whole body weight and body scale weight was on average -1.0 ± 0.7 kg and the interobserver TEM in our laboratory is 0.95% for the total mineral content, 1.52% for total body fat mass, 0.68% for lean soft tissue, and 0.86% for ALST.

### 2.4. Skeletal Muscle Mass Estimation

The ALST (kg) measured with DXA was converted to SMM (kg) with the equation published by Kim et al. [8]:

(1) SMM=1.13∗ALST–0.02∗Age+0.61∗Sex+0.97

where age is in years and sex is 1 for men and 0 for women (Table 1). The calculated SMM was set as the reference. Nine mathematical equations that estimate SMM through anthropometric measurements were analyzed (Table 1). Two equations from the Kuriyan's et al. [18] study were analyzed, one with corrected arm muscle area (CAMA) and the other with the arm muscle area plus the thigh muscle area (AMA/TMA).

### 2.5. Statistics

In order to analyze the variables' normal distribution, we performed the D'Agostino-Pearson omnibus normality test. The variables with normal distribution were expressed as mean ± standard deviation (SD); otherwise, the variables were expressed as median [25^th^–75^th^ percentile].

The SMM homogeneity of variances was determined by Bartlett's test, demonstrating significant differences between them (*p*<0.001). Therefore, to compare the SMM obtained between the equations and DXA, we performed a Kruskal-Wallis test using Dunn's test as* post hoc*. All results were deemed significant at a p value <0.05.

We calculated the mean difference (equation – DXA) and the 95% limits of agreement as described previously [26] for all the analyzed equations. Negative results represented underestimation while positive results of the analyzed equation represented overestimation. We also calculated the absolute range between the 95% limits of agreement and the Pearson coefficient of correlation (differences vs DXA).

The statistical analyses were performed with the software GraphPad Prism® version 7.04 for Windows® (GraphPad Software Inc., La Jolla, California, USA).

## 3. Results

### 3.1. Participants and Body Composition

The sample mean age was 24.4 ± 4.6 years. They showed 74.5 ± 7.8 kg of body weight, 177.7 ± 6.1 cm of height, and 23.6 ± 1.8 (kg/m^2^) of body mass index. The body composition components measured with DXA were on average 14.9 ± 3.2 for body fat percentage, 62.5 ± 5.9 kg for lean body mass, 28.1 ± 2.9 kg for ALST, and 32.4 ± 3.4 kg for SMM. The subjects' anthropometric measurements associated with the SMM prediction are listed in Table 2.

### 3.2. Anthropometric Equations

**Figure 1:**
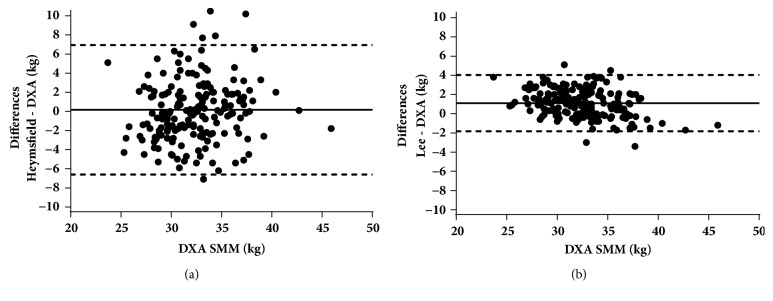
Bland-Altman plots for Heymsfield (a) and Lee (b) equations compared with DXA. Solid line represents mean differences; dashed lines represent 95% confidence intervals (mean ± 2SD). One value is hidden on (a) plot (x = 35.3; y = 14) because of scale adjustment. DXA: dual energy x-ray absorptiometry; SMM: skeletal muscle mass.

From the nine equations analyzed, Heymsfield's [12] and Lee's [17] were statistically similar to DXA-derived SMM values (Table 3). Heymsfield's equation had a mean difference of -0.17 kg, and its limits of agreement ranged from -6.61 to 6.94 kg. Lee's equation had a mean difference of 1.10 kg, and its limits of agreement ranged from -1.83 to 4.03 kg (Table 4, Figure 1). The other equations showed higher mean differences and wider limits of agreement compared with DXA. The equations that overestimated the most were those reported by Martin et al. [15] and Doupe et al. [16] (Table 3). Conversely, from the equations that differed significantly from DXA, those reported by Martin et al. [15] and Kuriyan et al. [18] (CAMA) showed the widest limits of agreement (Table 4).

## 4. Discussion

In our study, we found that the SMM estimated with the equations from Heymsfield [12] and Lee [17] were statistically similar to values obtained with DXA. The equation published by Heymsfield et al. [12], had the smallest mean difference but also showed wider limits of agreement (13.6 kg range) (Table 4). This variability could be explained because Heymsfield's equation uses only two predictor variables (Table 1) and because of the differences to estimate SMM between the Heymsfield's reference method (urine creatinine) and ours (DXA).

The SMM estimated with the equation published by Lee et al. [17] had a small mean difference with DXA and narrower limits of agreement (5.9 kg range) (Table 4). This smaller variability could be attributable to the use of more variables (three limb girths: relaxed arm, mid-thigh, and calf, corrected by skinfolds, Table 1) and the use of an anatomical cylindrical model [8, 12].

Other studies have compared the accuracy of anthropometric equations to estimate SMM in different populations. For example, Berral de la Rosa et al. [22] compared the validity of six anthropometric equations in 37 male badminton players. However, they compared the sum of several anthropometric equations to estimate different tissues (i.e., muscle mass, body fat, and bone) with the measured body weight, which is not an actual assessment of SMM. Rodriguez-Rodriguez et al. [23] compared five anthropometric equations in 20 nonprofessional cyclists. The SMM measured with DXA was the reference method in a similar manner to that in our study. They reported that the equations published by Heymsfield [12] and Drinkwater [13] were statistically similar to DXA-derived values; however, they did not perform a deeper statistical analysis for the agreement between these equations and DXA. A few years earlier, Gobbo et al. [19] analyzed four anthropometric equations in 131 male college students. They also used DXA-derived SMM values as the reference method and reported that Lee's equation [17] was the most accurate in their sample. Finally, Rech et al. [20] evaluated 120 women and 60 men aged 60 years and older, and they compared three anthropometric equations using DXA-derived SMM values as the reference. They found in their sample that Lee's equation [17] was the most accurate to estimate SMM. Findings in these studies are similar to those reported here, where we observed that the equation published by Lee [17] was the most accurate to estimate DXA-derived SMM [19, 20] and that Martin's [15] and Doupe's [16] equations significantly overestimate SMM [19, 20, 22, 23] (Tables 3 and 4).

The importance of SMM for exercise performance is well recognized [4, 5, 27]; however, it is difficult to find studies that report SMM, probably because it is difficult to assess it accurately, requiring expensive and complex methods such as magnetic resonance, computed axial tomography, or DXA.

However, we found that, among professional male soccer players, SMM can be accurately estimated through Heymsfield's [12] and Lee's [17] equations (Table 4, Figure 1).

Some limitations in our study were as follows: (1) the ALST was measured and analyzed with different equipment and software (Hologic) than the one used by Kim et al. [8] (Lunar); (2) the skinfolds were measured with a Harpenden caliper, different to the equations that used Lange (Lee [17]; Heymsfield [12]) and Holtain (Kuriyan [18]) calipers; (3) the anatomical sites employed for the anthropometric measurements in some of the equations were not exactly as how we located them according to our standardized procedure [24, 25], but as closest as possible to the original site; and (4) the equations analyzed in this study were developed with different reference methods such as magnetic resonance imaging (Lee [17]), urine creatinine (Heymsfield [12]; Kuriyan [18]), cadaver dissection (Drinkwater [13]; Kerr [14]; Martin [15]; Doupe [16]), and remaining weight (De Rose [11]). These differences in the reference method account for the differences observed with the SMM obtained by DXA, because they estimate SMM at different levels of composition and with different assumptions [28].

## 5. Conclusion

We found that the SMM evaluated with DXA, in professional male soccer players, can be accurately estimated with the anthropometric equations published by Lee [17] and Heymsield [12]. Lee et al.'s equation was the best for estimating SMM. Conversely, the Heymsfield equation demonstrated greater variability and therefore should be used with caution. The use of these anthropometric equations to accurately assess changes in SMM among soccer players (or athletes) remains to be elucidated and deserves further research.

## Figures and Tables

**Table 1:** Analyzed anthropometric equations for estimating skeletal muscle mass.

Author	Equation
Kim (2002) [8]	*SMM* = (1.13 *∗ALST*) – (0.02 *∗ Age*) + (0.61 *∗ SexA*) + 0.97
De Rose (1980) [11]	*SMM = BW* – (*FM + BM + RM*)
Heymsfield (1982) [12]	*SMM = Height∗* [0.0264 + (0.0029 *∗CAMA*)]
Drinkwater (1980) [13]	*SMM* = (*AZ ∗* 2.99 + 25.55)/(170.18/*Height*)^3^
Kerr (1988) [14]	*SMM* = (*ZMU ∗* 5.4 + 24.5)/(170.18/*Height*)^3^
Martin (1990) [15]	*SMM = *[*Height∗* (0.0553 *∗CMTG*^2^ + 0.0987 *∗ FAG*^2^ + 0.0331 *∗CCG*^2^) – 2445]/1000
Doupe (1997) [16]	*SMM* = [*Height ∗* (0.031 *∗ CMTG*^2^* +* 0.064 *∗ CCG*^2^ + 0.089 *∗ CAGm*^2^) – 3006]/1000
Lee (2000) [17]	^a^ *SMM* = (*Height*/100) *∗* (0.00587 *∗CAG*^2^ + 0.00138 *∗CMTG*^2^ + 0.00574 *∗CCG*^2^) + (2.4 *∗SexA*) – (0.026 *∗ Age*) + *Race* + 4.4
Kuriyan (CAMA) (2008) [18]	*SMM* = 14.718 + (0.366 *∗CAMA*)
Kuriyan (AMA/TMA) (2008) [18]	*SMM* = 10.122 + (0.23 *∗ AMA*) + (0.049 *∗TMA*)
Apendix	^b^ *FM* = 0.01 *∗*{*BW ∗*[(*SbSF + TSF + SpSF + ASF*) *∗* 0.153 + 5.783]}
	^b^ *BM* = 3.02 *∗* [(*Height*/100)^2^*∗* (*BSD*/100) *∗* (*BFD*/100) *∗* 400]^0.712^
	^b^ *RM = BW∗* 0.241
	*CAMA = AMA – SexB*
	*AMA = *{[*ARG* – (*π ∗ TSF*/10)]^2^/4*π*}
	*TMA = *{[*MTG* – (*π∗ThSF*/10)]^2^/4*π*}
	*ZMU = *[(*CAG + FAG + CMTG + CCG + CChG*) *∗* (170.18/*Height*)] – 207.21/13.74
	*CAG = ARG* – (*π ∗ TSF*/10)
	*CAGm = ARG – *{*π ∗*[(*Average of TSF + BSF*)/10]}
	*CMTG = MTG – *(*π ∗ ThSF*/10)
	*CTG = TG – *(*π ∗ ThSF*/10)
	*CCG = CG – *(*π ∗ CSF*/10)
	*CChG = ChG – *(*π ∗ SbSF*/10)
	*AZ = *(*ZCAG + ZCTG + ZCCG + ZCChG*)/4
	*ZCAG* = 1/1.91 *∗* [*CAG∗* (170.18/*Height*) – 22.05]
	*ZCTG* = 1/3.59 *∗* [*CTG ∗*(170.18/*Height*) – 47.34]
	*ZCCG* = 1/1.97 *∗* [*CCG∗* (170.18/*Height*) – 30.22]
	*ZCChG* = 1/4.86 *∗* [*CChG∗* (170.18/*Height*) – 82.46]

^a^Race was hispanic for all the samples.

^b^Equations listed as reported by De Rose (1980) [11].

Abbreviations: Age: years; ALST: appendicular lean soft tissue (kg); AMA: arm muscle area (cm^2^); ARG: arm relaxed girth; ASF: abdominal skinfold; AZ: Z-score average; BFD: biepicondilar femur diameter (cm); BM: bone mass (kg); BSD: bi-stylion diameter (cm); BSF: biceps skinfold; BW: body weight (kg); CAG; corrected arm girth; CAGm: corrected arm girth modified; CAMA: corrected arm muscle area (cm^2^); CCG: corrected calf girth; CChG: corrected chest girth; CG: calf girth; ChG: chest girth; CMTG: corrected mid-thigh girth; CSF: medial calf skinfold; CTG: corrected thigh girth; FAG: forearm girth; FM: fat mass (kg); height (cm); MTG: mid-thigh girth; race: Asian = -1.6, Afro-American = 1.2, White or Hispanic = 0; RM: residual mass (kg); SbSF: subscapular skinfold; SexA: men =1, women = 0; SexB: men = 10, women = 6.5; SMM: skeletal muscle mass (kg); SpSF: supraspinal skinfold; TG: thigh girth; ThSF: front thigh skinfold; TMA: thigh muscle area (cm^2^); TSF: triceps skinfold; ZCAG: Z-score of CAG; ZCCG: Z-score of CCG; ZCChG: Z-score of CChG; ZCTG: Z-score of CTG; ZMU: Z-score of sum of corrected girths.

**Table 2:** Participants' anthropometric measurements (n = 179).

Skinfolds (mm)^a^			
Triceps	8.0	[6.2 – 9.9]	(3.8 – 16.0)
Subscapular	9.8	[8.0 – 11.7]	(5.4 – 19.5)
Biceps	3.8	[3.2 – 4.8]	(2.3 – 8.0)
Supraspinal	8.1	[6.3 – 11.6]	(3.5 – 26.3)
Abdominal	15.9	[11.1 – 21.6]	(5.1 – 36.5)
Front Thigh	8.4	[7.0 – 11.0]	(3.0 – 21.3)
Medial Calf	5.3	[4.5 – 6.4]	(2.5 – 15.9)

Girths (cm)^b^			

Arm Relaxed	30.8	±2.1	(25.0 – 36.5)
Forearm	26.9	±1.3	(23.6 – 30.7)
Chest	96.7	±4.9	(86.0 – 112.5)
Thigh	58.1	±3.3	(47.7 – 67.4)
Mid-thigh	54.0	±3.2	(47.2 – 63.7)
Calf	36.7	±1.9	(31.8 – 41.5)

^a^Data expressed as median [25^th^ – 75^th^ percentile] (minimum – maximum).

^b^Data expressed as mean ± SD (minimum – maximum).

**Table 3:** Skeletal muscle mass (kg) obtained by anthropometric equations compared with DXA (n = 179).

Author	Mean ± SD	*p* value^a^
DXA	32.4 ± 3.5	-
Heymsfield (1982)	32.6 ± 5.2	0.99
Lee (2000)	33.5 ± 3.3	0.92
Kuriyan (CAMA) (2008)	34.5 ± 3.3	<0.001
De Rose (1980)^b^	34.5 ± 3.3	<0.001
Kuriyan (AMA/TMA) (2008)	35.1 ± 3.0	<0.001
Drinkwater (1980)	36.0 ± 4.1	<0.001
Kerr (1988)	35.8 ± 4.9	<0.001
Doupe (1997)	38.7 ± 5.1	<0.001
Martin (1990)	43.3 ± 5.5	<0.001

^a^Compared with DXA.

^b^n = 146

Abbreviations: DXA, dual-energy X-ray absorptiometry; CAMA, corrected arm muscle area; AMA, arm muscle area; TMA, thigh muscle area.

**Table 4:** Analysis of absolute (kg) skeletal muscle mass differences (equation – DXA) in professional male soccer players (n = 179).

	Author								
	Lee	Heymsfield	De Rose^a^	Drinkwater	Kuriyan (AMA/TMA)	Doupe	Kerr	Kuriyan (CAMA)	Martin
Mean difference	1.10	0.17	2.21	3.57	2.66	6.31	3.38	2.08	10.91
Mean + 2SD	4.03	6.94	5.19	7.27	7.01	11.26	8.46	7.48	16.63
Mean – 2SD	-1.83	-6.61	-0.76	-0.13	-1.70	1.35	-1.71	-3.32	5.19
r	-0.34^b^	0.14	-0.23^b^	0.15	-0.51^b^	0.47^b^	0.30^b^	-0.43^b^	0.50^b^
Range	5.9	13.6	6.0	7.4	8.7	9.9	10.2	10.8	11.4

^a^n = 146

^b^Significant correlation (p<0.05).

Abbreviations: CAMA, corrected arm muscle area; AMA, arm muscle area; TMA, thigh muscle area.

95% limits of agreement represented as mean + 2SD (upper) and mean – 2SD (lower).

## Data Availability

The data used to support the findings of this study are available from the corresponding author upon request.
